# Interdigitated Paralemniscal and Lemniscal Pathways in the Mouse Barrel Cortex

**DOI:** 10.1371/journal.pbio.0040382

**Published:** 2006-11-21

**Authors:** Ingrid Bureau, Francisca von Saint Paul, Karel Svoboda

**Affiliations:** Howard Hughes Medical Institute, Cold Spring Harbor Laboratory, Cold Spring Harbor, New York, United States of America; Vanderbilt University, United States of America

## Abstract

Primary sensory cortical areas receive information through multiple thalamic channels. In the rodent whisker system, lemniscal and paralemniscal thalamocortical projections, from the ventral posteromedial nucleus (VPM) and posterior medial nucleus (POm) respectively, carry distinct types of sensory information to cortex. Little is known about how these separate streams of activity are parsed and integrated within the neocortical microcircuit. We used quantitative laser scanning photostimulation to probe the organization of functional thalamocortical and ascending intracortical projections in the mouse barrel cortex. To map the thalamocortical projections, we recorded from neocortical excitatory neurons while stimulating VPM or POm. Neurons in layers (L)4, L5, and L6A received dense input from thalamus (L4, L5B, and L6A from VPM; and L5A from POm), whereas L2/3 neurons rarely received thalamic input. We further mapped the lemniscal and paralemniscal circuits from L4 and L5A to L2/3. Lemniscal L4 neurons targeted L3 within a column. Paralemniscal L5A neurons targeted a superficial band (thickness, 60 μm) of neurons immediately below L1, defining a functionally distinct L2 in the mouse barrel cortex. L2 neurons received input from lemniscal L3 cells and paralemniscal L5A cells spread over multiple columns. Our data indicate that lemniscal and paralemniscal information is segregated into interdigitated cortical layers.

## Abstract 2

Using laser scanning photostimulation, the authors map thalamocortical circuits in the mouse somatosensory cortex at an unprecedented level. They reveal a functional segregation of thalamic pathways between distinct cortical layers that is maintained within the intracortical circuitry as well.

## Introduction

The large facial whiskers map somatotopically onto the barrel cortex of rodents [1]. Layer (L)4 neurons are arranged into clusters (barrels) that can be used to visualize the sensory map [1,2]. Between barrels are the septa [1,3]. The L4 barrels and septa are landmarks that define functional columns spanning all cortical layers. Neurons in each barrel and directly above and below the barrel (barrel-related column) are excited by the stimulation of a particular whisker with short latencies, and more weakly by its neighbors [4–7]. Neurons aligned with septa respond to multiple whiskers with longer latencies [5,8]. In the rat somatosensory system, barrels and septa are associated with different thalamocortical [9–11] and intracortical [12,13] circuits. In the mouse barrel cortex, septa are small and cell-poor [1,14], raising the possibility of qualitative differences between the intracortical circuits in the rat and mouse.

The barrel cortex receives two types of thalamocortical input, lemniscal and paralemniscal, which are relayed through the ventral posteromedial nucleus (VPM) and the posterior medial nucleus (POm), respectively [15,16]. The activity of POm neurons may primarily encode information about whisking, whereas VPM neurons may encode combined information about whisking and whisker–object contact [17]. VPM projection neurons send axons to cortical layers L3, L4, L5B, and L6A of the barrel-related columns, with the highest axonal density in L4 [9–11]. POm projection neurons send axons mainly to L5A and L1 as well as to L4 septa. However, since pyramidal cell dendrites span multiple layers, neurons in L2/3, L5A, L5B, and L6 are all potential targets for both VPM and POm.

In vivo measurements of adaptation to repetitive whisker deflections suggest that L4 and L5B neurons share features with lemniscal responses recorded in VPM, whereas L5A neurons share similarities with paralemniscal responses recorded in POm [15,18]. In contrast, mapping experiments show that L5A neurons have sharp receptive fields, typical in the lemniscal pathway, whereas L5B neurons have broad receptive fields, suggestive of the paralemniscal pathway [19]. Complicating the interpretation of these measurements is the fact that cortical receptive fields are shaped by thalamocortical projections in cooperation with intracortical circuits [20]. In addition, POm neurons are difficult to excite by whisker stimulation in anesthetized animals [21,22]. Taken together, these anatomical and functional studies suggest that lemniscal and paralemniscal pathways may be segregated in distinct thalamocortical and intracortical circuits, but the detailed circuit diagram is poorly understood.

Here we used two types of brain slice preparation to map thalamocortical and intracortical circuits in the mouse somatosensory system in vitro. Since POm and VPM cell axons are intermingled in the thalamus [9,11] and only separate after arriving in the cortex, stimulating electrodes are difficult to use to selectively excite axons originating in the VPM or POm. Instead, we used laser scanning photostimulation (LSPS) [13,23–26], which stimulates neurons close to their cell body, without exciting axons of passage. We find that lemniscal and paralemniscal thalamocortical projections are segregated in different cortical laminae. We also describe intracortical projections to functionally distinct supragranular layers: L3 cells receive spatially focused lemniscal inputs from L4, whereas L2 cells integrate lemniscal and paralemniscal input over multiple columns.

## Results

### Mapping Thalamocortical Circuits Using LSPS

**Figure 1:**
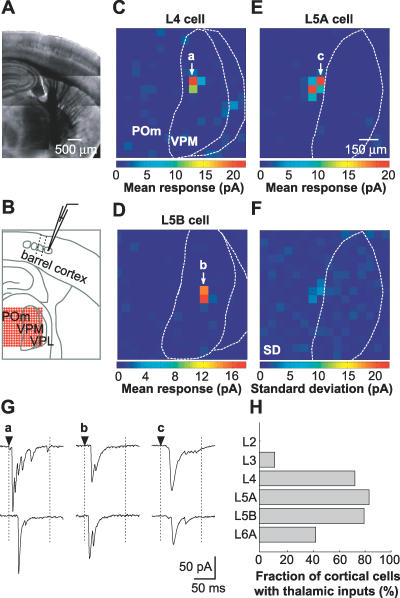
Laser Scanning Photostimulation Mapping of Thalamocortical Projections (A) Montage of a thalamocortical slice. (B) Layout of the experiment. Excitatory neurons were recorded in the barrel cortex while thalamic neurons were photostimulated by glutamate uncaging. The map pattern (red grid) was centered on the POm/VPM boundary. Dashed lines indicate a barrel-column. (C–E) Examples of synaptic input maps for individual L4 (C), L5B (D), and L5A (E) neurons. The pixel values encode the mean amplitudes of EPSCs measured within 100 ms after the stimulus (see [G]). The dashed lines indicate the borders between POm, VPM, and the ventral posterolateral nucleus (VPL) (see [B]). Letters mark a pair of pixels corresponding to the traces shown in (G). (F) Map of standard deviations across trials for an individual cell (same cell as in [E]). (G) Examples of individual EPSCs. The responses were evoked by photostimulation at the sites indicated by letters in (C–E) (two sites per arrow). Arrowheads indicate the timing of the stimulus; dashed lines indicate the averaging window used for analysis. (H) Percentage of cortical cells with thalamic input. The percentage was computed for cells that were within a lateral distance of 300 μm of cells that showed thalamic input in the brain slice.

We prepared barrel cortex thalamocortical slices from young (postnatal day 12–18) mice. Under brightfield illumination, VPM appeared as a dark, kidney-shaped structure [2] (Figure 1A and 1B). POm appeared as a bright region on the medial side of VPM [27]. Barrels and septa in L4 of the neocortex could also be recognized [27]. Whole cell voltage-clamp recordings were made from excitatory neurons in L4 barrels or in infragranular or supragranular layers centered on barrels (i.e., in barrel-related columns) (Figure 1B–G). Excitatory postsynaptic currents (EPSCs) were isolated by holding neurons near the reversal potential for inhibition (−70 mV). Simultaneously, glutamate was photoreleased in the focal spot of an ultraviolet (UV) laser beam on a 16 × 16 pixel grid (75-μm spacing). The grid covered POm and VPM, with its center aligned to the POm/VPM boundary (Figure 1B).

Photostimulation by glutamate uncaging in the thalamus evoked brief (duration < 100 ms) bursts of EPSCs (range 1–10) in neocortical neurons (Figure 1G). EPSCs triggered by VPM neurons had faster rise and decay times than those from POm neurons (Table S1). Although their sizes varied, the amplitudes and temporal profiles of the EPSCs suggested that they were caused by only a few thalamic cells (Figure 1G). Because of the small size of these EPSCs, it was unlikely that they triggered action potentials (APs) in neocortical neurons. This was verified by recording APs using loose-seal recordings in neocortical neurons. Recordings were restricted to columns in which thalamic input had been detected. In all cases, photostimulation failed to trigger APs in the neocortex (*n =* 9). Therefore, EPSCs evoked by photostimulation in thalamus arise at thalamocortical synapses.

Responses were quantified as the average synaptic current during a 100-ms window immediately after the UV flash (Figure 1G; see Materials and Methods). The maps of synaptic input show the spatial distribution of the origins of excitatory inputs received by individual neocortical neurons. Repeated maps for individual cells were highly consistent (Figure 1F) [28]. The thalamic regions where photostimulation evoked synaptic currents in cortical cells were small, including only a few (one to five) uncaging sites (i.e., pixels), which were almost always contiguous (Figure 1C–1E).

About 50 % (111/223) of the recorded cells showed thalamic input. However, this underestimated the fraction of cortical neurons receiving thalamic input because thalamocortical axons were partially severed in the brain slice, and because only the most superficial third of the brain slice is accessible to photostimulation [29]. Indeed, if one neuron in a particular barrel column responded to thalamic stimulation, a second neuron within a lateral distance of 300 μm was also likely to respond (70% [32/44] in L4; 80% [48/57] in L5A; 80% [16/20] in L5B; and 40% [6/14] in L6A) (Figure 1H). This suggests that the vast majority of excitatory neurons in L4–L5B receive thalamic inputs in vivo.

In contrast, few pyramidal cells recorded in L2/3 (0/9 in L2, 70–125 μm below the pia; and 2/17 in L3, >125 μm below the pia) displayed synaptic input from the thalamus (VPM). The lack of input is unlikely due to slicing artifacts. First, all L2/3 cells were recorded in columns in which thalamic inputs were detected in L4 or L5A. Second, the chance of recording thalamic inputs did not vary monotonically with cortical layer (Figure 1H). Third, to further reduce the possibility of slicing artifacts, about half (14/26, seven L3 and seven L2 cells) of the L2/3 neurons tested were recorded in brain slices that were cut parallel to the plane of dendrites and axons in cortex (Materials and Methods). Our results suggest that the thalamic innervation of L2/3 cells is sparse.

### Spatial Resolution of LSPS in Thalamus

**Figure 2:**
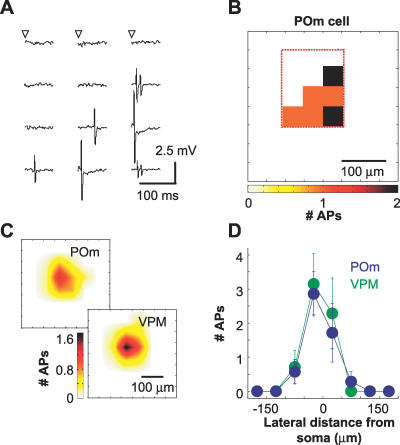
Spatial Resolution of LSPS in the Somatosensory Thalamus (A) Photostimulation-evoked APs recorded in loose-patch mode in a POm cell (arrowheads indicate the stimulus). The traces correspond to the 12 pixels in the boxed region shown in (B). (B) Excitation profile of a single POm cell. The grid was centered on the soma. Pixel values encode the number of APs in a 100-ms window after photostimulation. (C) Average excitation profiles (VPM, *n =* 7; POm, *n =* 7). (D) Excitation as a function of lateral distance from the soma (at 0). Responses elicited in each column of the 8 × 8 grid were summed.

Interpreting input maps in terms of thalamocortical topography requires characterization of the spatial resolution of LSPS mapping. At a particular spot (i.e., pixel) in the synaptic input maps (Figure 1C–1E), the amplitude of the postsynaptic response is proportional to the number of excited thalamic neurons (*N*_cell_), the number of APs fired per stimulated neuron (*N*_AP_), and the average strength of the synaptic connection (*q*_con_): Pixel value ∝ *N*_cell_ *N*_AP_ *q*_con_. To quantify the spatial resolution of LSPS in the thalamus, we directly measured *N*_AP_ as a function of distance from the soma for VPM and POm cells in loose patch recordings (Figure 2A and 2B) (“excitation profiles”; pixel grid, 8 × 8; pixel spacing, 50 μm). VPM and POm cells had similar excitation profiles (Figure 2C and 2D). Upon photostimulation, thalamic cells fired one to five APs with delays on the order of 20 ms (Table S1). Thalamic cells fired only when uncaging occurred close to their cell body (Figure 2D), at a mean lateral distance of 32 ± 2 μm (VPM) and 41 ± 6 μm (POm) (see Materials and Methods). These distances imply that synaptic inputs evoked in cortical neurons by photostimulating approximately 35 μm on either side of POm/VPM boundary can be reliably assigned as originating from a particular thalamic nucleus. The LSPS resolution is similar in the barrel cortex (see Materials and Methods).

### Laminar Organization of VPM and POm Projections

**Figure 3:**
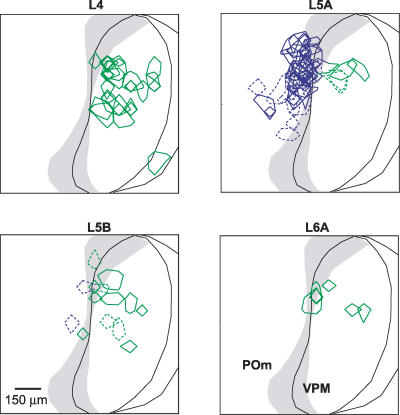
Laminar Organization of Thalamocortical Projections Overlay of input domains for L4, L5A, L5B, and L6A cells. The contours enclose regions where the inputs exceed 50% of the largest responses (VPM, green; POm, blue). Dashed contours correspond to pyramidal cells with apical dendrites that were cut below L1. Shaded area denotes the range of POm/VPM boundaries for all experiments.

For each recorded cortical neuron, we defined a thalamic “input domain” as the region containing responses larger than half of the maximum response (see Materials and Methods). We then overlaid input domains from different brain slices, recorded in diverse barrel-related columns, on a template of the brain slice. Most cortical cells had a single contiguous input domain in POm or VPM. A small fraction (3/42) of L5A neurons had two domains. Cortical cells could be clearly separated into two groups according to the locations of their input domains: L4, L5B, and L6A cells received mainly VPM input, and L5A cells received mainly POm input (Figure 3).

Input domains for L4 cells occupied the dorsal part of VPM (27/28). Since this region is known to be innervated by cells from the principal trigeminal nucleus [30,31], the thalamic cells that project to L4 belong to the lemniscal pathway. The input domains of L5B (11/13) and L6A (6/6) cells also mostly occupied the dorsal VPM. One L5B cell had an input domain straddling the POm/VPM border, whereas a second cell had an input domain in POm only (blue contours). Since these cells were far from the L5B/L5A border, and thus unambiguously in L5B, these findings imply that L5B neurons receive sparse POm input in addition to VPM input.

The input domains for L5A cells were mostly (38/42) confined to a narrow band in the lateral part of POm, close to the border with VPM (Figure 3). In our sample, one L5A cell had an input domain straddling the POm/VPM border, and for three others, the input domains were in VPM. These findings imply that L5A cells are dominated by POm input, but can also receive sparse VPM input.

L5B and L5A pyramidal cells have apical dendrites that span L1–L5. Because POm cells send axonal branches to L1 [9,32], it is possible that they make synapses with L5A and L5B cells in this layer. However, both of the L5B cells receiving POm input had their apical dendrites cut in L4 (Figure 3, dashed contours), implying that the POm axons innervated these cells on their basal dendrites. L5A cells were also innervated by POm cells on their basal dendrites. Indeed, L5A cells with cut and intact apical dendrites had POm inputs with indistinguishable amplitudes (intact: 5.6 ± 0.8 pA, *n =* 16; cut 6.3 ± 0.9 pA, *n =* 19). This implies that our mapping experiments report thalamocortical synapses on the basal dendrites of L5 neurons. It is possible that POm synapses terminating in L1 are too weak to make a large contribution to EPSCs recorded in the soma or that POm axons in L1 synapse onto postsynaptic neurons that were not recorded in our study.

### Input Domains Projecting to Individual Cortical Columns

**Figure 4:**
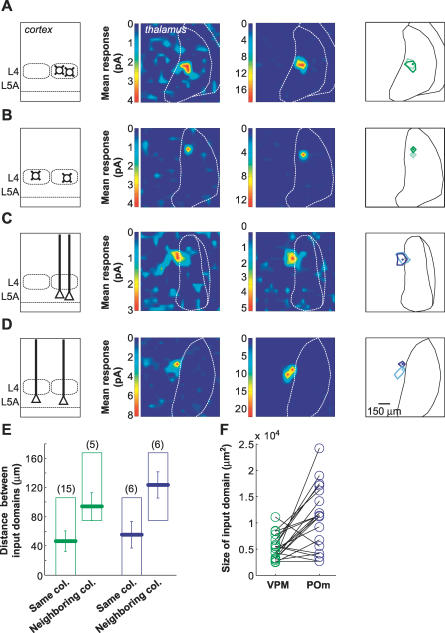
Thalamic Input Domains Projecting to Individual Cortical Columns (A–D) Schematic of the locations of recorded neurons (left), input maps (middle), and overlaid input domains (right) Circles in the input domains indicate the largest responses from a pair of L4 cells in the same column (A), a pair of L4 cells in neighboring columns (B), a pair of L5A cells in the same column (C) and a pair of L5A cells in neighboring columns (D). (E) Distance between the largest input in the synaptic input maps (green, VPM; blue, POm) from pairs recorded in the same column or in neighboring columns. The average (thick line), and the minimum and maximum distances between largest input across cells (rectangle) are shown. Numbers in parenthesis are the number of pairs. The pairs of cells located in the same column were three L4/L4, five L4/L5B, four L4/L6A, two L5B/L6A, one L6A/L6A, and six L5A/L5A. Pairs of cells located in neighboring columns were four L4/L4, one L4/L5B, and six L5A/L5A cells. (F) Areas of the input domains in VPM (green) and POm (blue) for pairs of L4/L5B/L6A, and L5A cells located in the same column.

To estimate the precision of thalamocortical maps, we compared input maps for pairs of cells within the same cortical columns or in neighboring columns (Figure 4). Pairs of VPM-recipient cells located in the same column (L4/L4, L4/L5B, and L4/L6A) had overlapping input domains (Figure 4A). The distance between the largest inputs was small (average distance, 47 ± 14 μm; *n =* 15; Figure 4E), on the order of the resolution limit of our technique. Pairs of cells with VPM input located in neighboring columns had input domains that were offset vertically (Figure 4B). For these pairs, the distance between the largest inputs was about 2-fold larger than for input domains in the same column (average distance, 94 ± 19 μm; *n =* 5; Figure 4E). These distances are consistent with the dimensions of barreloids, which have an ellipsoidal shape with diameters of approximately 50 and 120 μm, respectively [33,34].

Similar experiments were performed for pairs of L5A neurons with nearly identical results (Figure 4C and 4D). The distance between the largest inputs in POm was small for pairs recorded in the same column (average distance, 55 ± 18 μm; *n =* 6; Figure 4E) and larger for pairs recorded in neighboring columns (average distance, 124 ± 18 μm; *n =* 6; Figure 4E). These results suggest that the spacing between thalamic regions projecting to neighboring barrel columns is on the order of 100 μm and similar in VPM and POm.

We next compared the sizes of VPM and POm input domains of cortical cells recorded in the same barrel-related column. The sizes of the input domains for VPM-recipient neurons in L4, L5B, and L6A cells, recorded in the same column, were similar (ratio of input domain areas: L5B/L4, 0.85 ± 0.14, *n =* 5; and L6A/L4, 0.96 ± 0.26, *n =* 4). This suggests that the precision of the mapping of VPM onto cortex is similar in L4, L5B, and L6A. In contrast, the input domains in POm were larger than in VPM (ratio of input domain areas, 2.37 ± 0.39; range: 0.5–7.5, *n =* 20, *p* < 0.01, Mann-Whitney) (Figure 4F). Because POm and VPM cells have similar excitation profiles (Figure 2D) (i.e., they fire the same number of APs at a similar distance from their somata upon glutamate uncaging), the larger POm input domains imply that POm-recipient cells potentially receive input from a larger number of thalamic neurons, distributed over a larger thalamic volume, than VPM-recipient cells.

### Thalamocortical Topographic Transformations

**Figure 5:**
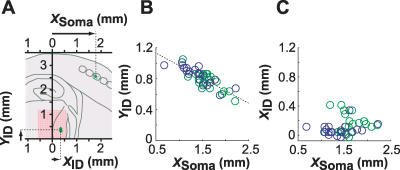
Thalamocortical Topographic Transformations (A) Schematic of the coordinate system. *X*_ID_ and *Y*_ID_ are the horizontal and vertical coordinates of the input domain, respectively. *X*_Soma_ is the horizontal coordinate of the cell position in cortex. *X*_ID_ and *X*_Soma_ are measured from the VPM/POm boundary (center of the uncaging grid; light red). *Y*_ID_ is measured from the bottom of VPM (bottom of the uncaging grid). (B) and (C) Positions of the input domains (*Y*_ID_ in B_;_ *X*_ID_ in C) in VPM (green) and POm (blue) as a function of *X*_Soma_. Cells were in L4 (*n =* 12); L5B (*n =* 7); L6A (*n =* 5); and L5A (*n =* 21). Dashed line shows the linear correlation between *X*_Soma_ and *Y*_ID_ for VPM and POm domains (*r^2^* = 0.72).

To explore the somatotopic organization of thalamocortical projection in the brain slice, we measured the locations of VPM and POm input domains (horizontal, *X*_ID_; vertical, parallel to the POm/VPM boundary, *Y*_ID_) relative to the horizontal location of the cortical cells (*X*_Soma_) (Figure 5A). A horizontal shift (increasing *X*_Soma_) corresponded to vertical downward shift (decreasing *Y*_ID_) for both VPM (*n =* 24) and POm (*n =* 21) input domains (linear correlation, *r^2^* ∼ 0.70) (Figure 5B). In contrast, *X*_Soma_ was not correlated with *X*_ID_ (Figure 5C) (*r^2^* < 0.20). These experiments confirm that in our thalamocortical slices, the somatosensory cortex and thalamus were cut across arcs and along rows [11,33,35].

**Figure 6:**
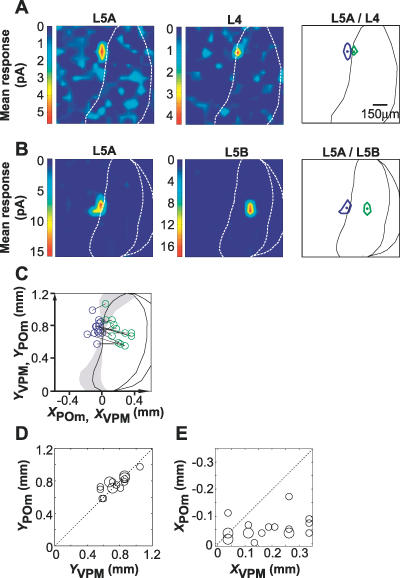
Somatotopic Maps in VPM and POm (A) Synaptic input maps (left) for a pair of L5A and L4 cells recorded in the same barrel-related column, and the overlaid input domains (right). Circles indicate the position of the largest responses in the input domains. (B) Same as (A) for a pair of L5A and L5B cells. (C) Positions of the centers of POm (blue) and VPM (green) input domains for pairs of cells recorded in the same column. (D) and (E) Distances of the centers of the input domains from the bottom of VPM (*Y*_POm_, *Y*_VPM_; [D]) and from the POm/VPM boundary (*X*_POm_, *X*_VPM_; [E]) for pairs of cells recorded in the same column (coordinate system is indicated in [C]). Larger symbols indicate overlay of multiple pairs (L5A/L4, *n* = 13; L5A/L5B, *n* = 5; L5A/L6A, *n* = 2). Dashed lines indicate the predicted relationships if VPM and POm somatotopy was mirror symmetric.

We next compared the locations of VPM and POm input domains projecting to the same cortical column in the same slice (Figure 6A and 6B). Pairs of cells (*n =* 20) were recorded in L5A and L4/L5B/L6A. The VPM and POm input domains had similar spatial distributions along the POm/VPM boundary (Figure 6C and 6D), indicating the existence of a somatotopic map in POm [22,36–38]. However, the distributions of the locations of VPM and POm input domains perpendicular to the POm/VPM boundary differed: the POm input domains were all close to the POm/VPM boundary (average distance from POm/VPM boundary, 53 ± 9 μm), whereas VPM input domains were scattered across the dorsal half of VPM (177 ± 24 μm; Figure 6C and 6E). This suggests that the somatotopic map in POm is compressed across whisker rows compared to the somatotopic map in VPM.

### Interdigitated Intracortical Relays

**Figure 7:**
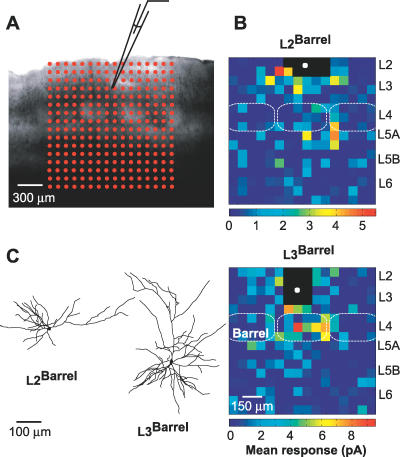
Laser Scanning Photostimulation Mapping of Intracortical Projections to L2/3 neurons (A) Across-barrel slice showing barrels corresponding to rows A–E (from left to right) under brightfield illumination. In this experiment, the map pattern (red grid) was centered on the septum between the barrels C and D. (B) Examples of synaptic input maps for a L2^Barrel^ cell (top) and a L3^Barrel^ cell (bottom). Black pixels are sites where glutamate uncaging evoked direct responses in the recorded cells, polluting the synaptic responses. Dashed lines indicate the barrel positions. Solid white circles show the cell body positions of the recorded neurons. (C) Examples of dendritic morphologies of L2^Barrel^ and L3^Barrel^ cells (same cells as in [B]).

Our mapping studies suggest that lemniscal projections from VPM target excitatory neurons mainly in L4, L5B, and L6A, whereas paralemniscal projections from POm target L5A. To trace the continuation of these circuits through the cortex, we used LSPS to map projections impinging on L2/3 pyramidal cells (Figure 7A and 7B) (see Materials and Methods; [13,28,39]).

**Figure 8:**
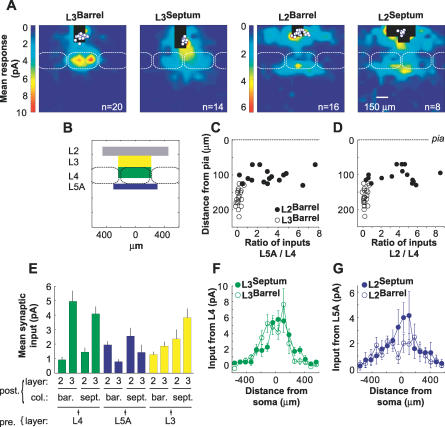
Interdigitated Intracortical Relays (A) Average synaptic input maps for positionally defined L2/3 pyramidal cells. (B) Regions of interest used for the analysis in (C–E). (C) The ratio of input from L5A/L4 as a function of the depth of the soma of barrel-related neurons. Dashed black line (top) indicates the pia. (D) The ratio of input from L2/3/L4. (E) Average synaptic input for projections defined by the presynaptic layer, postsynaptic layer, and postsynaptic column. For L3 neurons in septum and barrels, L4 input is significantly stronger than L5A input; for L2 neurons in barrels, L5A input is significantly stronger than L4 input (*p* < 0.005, Wilcoxon). Note that this pattern differs from that measured in the rat using essentially identical techniques (see Figure 3e in [13]). L3 input is significantly stronger for L2/3^Septum^ cells than for L2/3^Barrel^ cells (*p* < 0.05, Wilcoxon). (F) Horizontal profiles of L4 input to L3 cells. (G) Horizontal profiles of L5A input to L2 cells.

Deeper L2/3 cells had typical pyramidal morphology (Figure 7C) [29,40] and received input mainly from a locus in L4 immediately below the recorded neurons, and weaker input from L5A (Figure 7B). Neurons in the most superficial region of L2/3, 70–125 μm from the pia, had short apical dendrites or horizontal apical dendrites [41,42] (Figure 7C). They mainly received input from L5A and other L2/3 cells, and only weak input from L4 (Figure 7B). Measurements of excitation profiles of L4 and L5A neurons at the L4/L5A boundary revealed that the peak of input originating apparently in the lower part of L4 (Figure 8A, right) was likely due to the excitation of L5A pyramidal cells located close to the L4/L5A boundary (Figure S1). The ratios of input from L2, L4, and L5A were dramatically different for deep and superficial L2/3 cells (Figure 8B–8E) (*p* < 0.001, Wilcoxon), even for groups of neurons recorded in the same slice (Figure S2). On the basis of these circuit properties, we denote the superficial band of neurons (70–125 μm below the pia) as L2, and the remainder (125 μm below the pia to the barrels) as L3.

L2 and L3 cells also differed in terms of the horizontal distribution of their input. Although the L4→L3^Barrel^ projection was largely contained within one column (mean lateral distance from the center of map, 157 ± 6 μm, *n =* 20; see Materials and Methods), the L5A→L2^Barrel^ (235 ± 9 μm, *n =* 16) and L2/3→L2^Barrel^ (276 ± 11 μm, *n =* 16) projections were significantly wider (*p* < 0.001, Wilcoxon) and invaded neighboring columns (Figure 8A, 8F, and 8G).

In the rat, neurons above barrels and septa have strikingly different input maps (Figure 3e in reference [13]). Above barrels, L2/3 neurons receive strong input from L4 and little input from L5A. In contrast, above septa, L2/3 neurons are only weakly coupled to L4. L2 neurons receive strong input from L5A.

These differences are much less pronounced in the mouse. L3 cells above septa received strong input from L4, similar to L3 cells above barrels (Figure 8A, 8E, and 8F). L2 cells above barrels received strong input from L5A, similar to L2 cells above septa (Figure 8A, 8E, and 8G). We also analyzed the spatial distribution of input from L4 and L5A to individual L2/3 cells. Neurons above barrels and septa integrated input from cortical regions directly below (Figure S3). The somatopic map in L2/3 therefore varies smoothly across septa.

Still, there were differences between the input maps of L2 and L3 cells located above barrels and septa. L2/3^Septum^ cells received stronger input from L3 than L2/3^Barrel^ cells (*p* < 0.05, Wilcoxon) (Figure 8A and 8E). L3^Barrel^ cells received a sharper focus of input from L4 than L3^Septum^ cells (Figure 8F). On average, L4 input originated 153 ± 7 μm and 176 ± 8 μm laterally from the soma of L3^Barrel^ and L3^Septum^ cells, respectively (*p* < 0.05, Wilcoxon; see Materials and Methods).

## Discussion

We used LSPS to map two thalamocortical circuits in the mouse somatosensory system. We found that lemniscal VPM projections and paralemniscal POm projections are segregated in distinct layers in the barrel cortex. We further mapped intracortical circuits downstream of VPM and POm projections. We find that in mouse barrel cortex, ascending lemniscal and paralemniscal intracortical circuits remain segregated in different laminae in L2/3.

### Interdigitated Thalamocortical Circuits

LSPS allowed us to excite thalamic and neocortical neurons close to their cell body, avoiding the axons of passage. In the thalamocortical brain slice preparation, we were thus able to selectively excite neurons in VPM or POm. VPM neurons mainly targeted L4, L5B, and L6A neurons, whereas POm neurons targeted L5A neurons. This selectivity was not absolute: a small fraction (∼10%) of L5B neurons received POm input, whereas some (∼10%) L5A neurons received VPM input. It is possible this selectivity increases further with developmental age [43].

Only the photostimulation in the dorsal part of VPM elicited synaptic responses in L4/L5B/L6A. This region of VPM contains the barreloids and is innervated by axons from the principal trigeminal nucleus (PR5) [30,44], the defining feature of the lemniscal pathway. Responses in L5A were evoked only with photostimulation in a narrow band of POm, adjacent to the medial edge of VPM. This is consistent with previous reports of whisker-evoked responses in this region [21,22]. This region is innervated by large multipolar neurons from the interpolaris division of the spinal trigeminal nucleus (SP5i) [30,31], the defining feature of the paralemniscal pathway. Only one cell in our sample received input from the ventrolateral sector of VPM, a region innervated by small-sized neurons in SP5i (extralemniscal pathway) [17,45].

The laminar interdigitation of thalamocortical projections encoding distinct stimulus properties could be a common feature of all sensory systems. For example, in the visual system of the macaque monkey, projections from the magnocellular and parvocellular laminae in the lateral geniculate nucleus, specialized for motion/contrast and shape/color processing respectively, are segregated in different cortical layers of V1 [46].

### Dense Thalamocortical Connectivity in Granular and Infragranular Layers

In our experiments in brain slices, most cortical neurons in L4–L5B (70%–80%) received thalamic input. Since the majority of thalamocortical axons are cut, and since only neurons in the top 100 μm of the brain slice are excited efficiently by LSPS [13], the actual connectivity is likely higher. We conclude that most, perhaps all, excitatory neurons in L4–L5B receive input from the thalamus. In contrast, thalamic input was rarely detected in L2/3 neurons. This dramatic selectivity of thalamocortical projections for the deep layers was not expected from previous anatomical studies [9,11,32,47].

The whisker-evoked responses of L5 cells have properties reflecting both their thalamocortical and intracortical circuits. Upon whisker deflection, the L5B pyramidal cells fire with remarkably short latencies, comparable to L4 stellate cells, and before L2/3 pyramidal cells [48]. This is consistent with our finding that the amplitude of VPM input was comparable in L5B and L4 (Figure S1), despite the fact that thalamocortical terminations are less dense in L5B than in L4 [9–11]. L5B receptive fields are broader than L4 receptive fields [19], but we found that their input domains in VPM had similar sizes. This suggests that intracortical L2/3→L5B projections also play a prominent role in shaping L5B response properties.

L5A pyramidal cells fire later [15,48], but have sharper receptive fields than L5B cells [19]. These cells are likely driven by the strong L4→L5A projections [49,50]. The respective roles of the direct thalamic inputs and the intracortical inputs to L5 pyramidal neurons in the context of sensory processing remain to be discovered.

### Topography of Thalamocortical Projections

We compared the topography of the thalamocortical projections from POm and VPM across whisker arcs. We measured the sizes and spatial arrangement of the input domains corresponding to multiple arcs of the barrel field. Individual barrel columns received VPM input from small thalamic regions (diameter of input domains, ∼80 μm). The input domains for neighboring barrel columns were separated by approximately 100 μm. This arrangement suggests that VPM projections to cortex have very little divergence [6,34,35,51–54].

In contrast, the input domains in POm were larger (diameter, ∼120 μm), but had the same spacing (∼100 μm). This suggests that the projections from POm to cortex are more diffuse than from VPM, consistent with the large axonal arborizations of some POm cells [32]. Given their large arbors, POm cell axons are more likely partially severed in brain slices, suggesting that our measurement of POm input domains probably underestimates their actual size. Thus, L5A cells in neighboring barrel columns receive input from overlapping sets of POm neurons.

Input domains corresponding to multiple rows were distributed across VPM, orthogonal to the POm/VPM boundary, but compressed and indistinguishable in POm (Figure 6C–6E). These observations open up the possibility that POm projections to the barrel cortex could have an anisotropic divergence (higher across rows than across arcs). An alternative interpretation of our data is that the representation of whiskers across rows is arranged perpendicular to the plane of our brain slice.

### Intracortical Lemniscal and Paralemniscal Circuits

Previous studies in rats have suggested that lemniscal and paralemniscal circuits remain segregated in ascending circuits within the neocortex [8,13]. L2 and L3 neurons in barrel-related columns receive strong input from L4 barrels [13,39,40,55,56], with a minor input from L5A. In contrast, L2 neurons in septum-related columns receive strong input from regions in L5A below septa [13]. L3 neurons in septum-related columns are only weakly coupled to intracortical circuitry in brain slices [28]. Thus lemniscal and paralemniscal projections to L2/3 are segregated in discrete interdigitating columns in the rat.

The intracortical arrangement of lemniscal and paralemniscal circuits was different in mice. L3 neurons received strong input from L4 barrels, both in barrel-related columns and above septa. The L4 septa are narrow in mice (∼20 μm between rows, [1]), smaller than the resolution of LSPS (∼35 μm; see Materials and Methods). Although we cannot distinguish the contributions of septum and barrel cells to the responses evoked over the septa, the resolution of LSPS is sufficient to show that L3^Septum^ cells received L4 inputs predominantly from barrels (Figure 8A and 8F). Therefore L3^Septum^ cells are primarily lemniscal in mice. Some quantitative features of the projections to L3 differed in barrel-related columns and septum-related columns, but these differences are much more subtle than in the rat [13]. It is therefore likely that the response properties of L2/3 cells are similar above barrels and septa. Our data also suggest that the whisker map changes smoothly across the boundaries between barrel columns in mouse L2/3.

Paralemniscal projections from L5A primarily targeted a superficial band of neurons located between 70 and 125 μm below the pia. Because of their distinct inputs, we propose that these neurons constitute a bona fide layer 2 (L2). Does a similar L2 exist in rats? In previous LSPS studies “L2” and “L3” were defined simply by dividing L2/3 into two layers of equal thickness [13]. Other studies defined L2 as the superficial third of L2/3 [8]. These definitions are arbitrary since there is no clear cytoarchitectural demarcation of the L2/L3 boundary in the barrel cortex. Since previous studies of L2/3 organization in the rat did not specifically analyze neurons close to the border of L1, it is possible that a superficial L2 similar to mice may also exist in rats.

**Figure 9:**
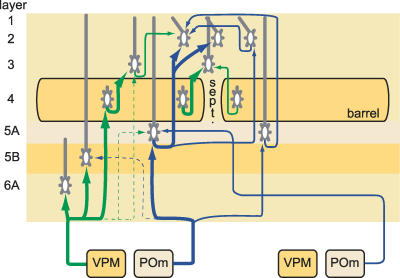
Circuit Diagram of the Thalamocortical and Ascending Intracortical Projections in the Barrel Cortex Lemniscal projections, green; paralemniscal projections, blue. Thick, thin, and dashed lines denote decreasing density of the projection.

The circuits feeding into L2 and L3 neurons further differ in their columnar organization. Although L3 neurons receive input from a sharp focus in L4, L2 neurons integrate input over multiple barrel columns. Furthermore, L2 neurons receive input from L3, suggesting that lemniscal and paralemniscal circuits converge in L2. We conclude that lemniscal and paralemniscal circuits are segregated in different thalamic nuclei and cortical laminae in mice: lemniscal, PR5 → VPM → L4/L5B/L6A → L3; paralemniscal, SP5i → POm → L5A → L2 (Figure 9). The functional importance of this precise interdigitation of thalamocortical and intracortical circuits remains to be determined.

## Materials and Methods

### Brain slice preparation and electrophysiology.

C57Bl6 mice were used in accordance with institutional guidelines. Thalamocortical slices (350 μm thick) (postnatal day [PND] 12–18) were prepared as described [2,57], with some modifications. A wide range of angles preserved thalamocortical projections to the barrel cortex. The angle between the blade and the midline was approximately 50°. The angle between the blade and the dorsal-ventral axis was typically 10°, but in some experiments, we used larger angles of 15°–45° to preserve apical dendrites. In brain slices cut parallel to dendrites, the angle between the slice plane and apical dendrites was close to 3°. Across-row barrel cortex slices (300 μm thick) (PND 17–23) were generated as described [13,58]. Slices were cut in chilled cutting solution containing (in mM): 110 choline chloride, 25 NaHCO_3_, 25 D-glucose, 11.6 sodium ascorbate, 7 MgCl_2_, 3.1 sodium pyruvate, 2.5 KCl, 1.25 NaH_2_PO_4_, and 0.5 CaCl_2_. Slices were then transferred to artificial cerebrospinal fluid (ACSF) containing (in mM): 127 NaCl, 25 NaHCO_3_, 25 D-glucose, 2.5 KCl, 1 MgCl_2_, 2 CaCl_2_, and 1.25 NaH_2_PO_4_, aerated with 95% O_2_/5% CO_2_. Slices were first incubated at 34 °C for 10–15 min and then maintained at room temperature prior to use.

Neurons (50–110 μm deep in the slice) were visualized with infrared gradient contrast optics and patched using borosilicate electrodes (4–6 MΩ). The intracellular solution contained (in mM) 128 K-methylsulfate, 4 MgCl_2_, 10 HEPES, 1 EGTA, 4 Na_2_ATP, 0.4 Na_2_GTP, 10 Na-phosphocreatine, 3 ascorbic acid, and 0.015 Alexa-594 (Molecular Probes, Eugene, Oregon, United States); (pH 7.25); 294 mOsm. Whole-cell recordings were made using a Multiclamp 700A (Axon Instrument, Molecular Devices, Sunnyvale, California, United States) amplifier. Cells were identified based on their laminar and columnar positions and their morphology, most often using the Alexa-594 fluorescence. The L3^Septum^ cells were within 2–49 μm (mean, 17 ± 3 μm) from a vertical line aligned with the septum center. L3 pyramidal cells had the following membrane properties: membrane capacitance (Cm) = 155 ± 5 pF; input resistance (Ri) = 225 ± 11 MΩ; and access resistance (Rs) = 24 ± 2 MΩ (*n =* 34). L2 cells: Cm = 134 ± 4 pF; Ri = 268 ± 15 MΩ; and Rs = 31 ± 2 MΩ (*n =* 24). L4 cells were stellate, with one exception, which was a pyramidal cell: Cm = 74 ± 3 pF; Ri = 501 ± 33 MΩ; and Rs = 35 ± 2 MΩ (*n =* 23). L5A was identified as a bright band under brightfield illumination, directly below the barrels in L4 (height, ∼100 μm). L5A cells were pyramidal: Cm = 168 ± 5 pF; Ri = 301 ± 17 MΩ; and Rs = 31 ± 1 MΩ (*n =* 42). L5B cells were pyramidal: Cm = 140 ± 8 pF; Ri = 282 ± 21 MΩ; and Rs = 32 ± 2 MΩ (*n =* 13). L6A cells were pyramidal: Cm = 94 ± 12 pF; Ri = 352 ± 45 MΩ; and Rs = 32 ± 2 MΩ (*n =* 6). All cortical neurons were likely excitatory. They were regular spiking and, in L2, L3, L 5, and L6, had pyramidal morphology. In parallel experiments, 65 L4 neurons were reconstructed, and all of these were spiny. Excitatory postsynaptic currents were measured in whole cell configuration at −70 mV. APs were recorded in loose-seal, cell-attached configuration. Custom software for instrument control and acquisition was written in Matlab (MathWorks, Natick, Massachusetts, United States).

### LSPS by glutamate uncaging.

LSPS was performed as described in [13]. Recirculating ACSF solution contained (in mM): 0.37 nitroindolinyl (NI)-caged glutamate (Sigma-Aldrich, St Louis, Missouri, Unites States; [59]), 0.005 (±)-3-(2-carboxypiperazin-4-yl)propyl-1-phosphonic acid (CPP), 4 CaCl_2_, and 4 MgCl_2_. Once whole cell recording was established, focal photolysis of caged glutamate was accomplished with a 1-ms pulse of a pulsed UV laser (wavelength, 355 nm; repetition rate, 100 kHz; DPSS Lasers, Santa Clara, California, United States) consisting of 100 pulses. Laser power was set to a power of 20 mW for LSPS in cortex and 20 or 30 mW for LSPS in thalamus. No significant difference was observed in the amplitudes of the strongest synaptic inputs or in the sizes of the input domains with 20 mW (64/86) or 30 mW (22/86).

The standard stimulus pattern for LSPS mapping consisted of 256 positions on a 16 × 16 grid. Spacing was set to 75 μm between adjacent rows and columns, giving a 1.125 × 1.125-mm mapping region. For the thalamocortical mapping experiments, the slice was oriented such that the internal capsule was vertical, the barrel cortex on the top. The grid of LSPS was centered on the POm/VPM boundary. The position of the grid was kept identical for all cells recorded in a slice. For each experiment, an image of the thalamus was acquired (Figure 1A). The boundaries between POm, VPM, and the ventral posterolateral nucleus of thalamus (VPL) were drawn using Neurolucida (MicroBrightField Bioscience, Williston, Vermont, United States). The standard shape of the POm/VPM boundary (black lines in Figures 3 and 6) is the mean of all boundaries, whereas the shaded area includes the range of all boundaries. Although the shape of the VPM nucleus was variable, it typically appeared as a bean-shaped structure with the concave side facing POm. For the LSPS in the barrel cortex, the vertical midline of the grid was centered on the barrel C or D, or in the septum between C and D. The horizontal midline was on top of the L4/L5A boundary.

Individual UV stimuli were presented every 1 s. Traces consisted of 100 ms of baseline prior to the stimulus, a 500-ms response interval, and a test pulse for measuring electrophysiological parameters. Excitation profiles of thalamic and cortical cells were generated under similar conditions except that cells were recorded in loose-seal configuration and glutamate was uncaged on a smaller pattern with 64 positions, centered on the soma (8 × 8 grid; 50 μm spacing; 350 × 350 μm).

### Analysis of LSPS data.

Thalamocortical input maps for individual neurons were constructed by computing the mean current amplitude calculated in a window of 100 ms immediately after the UV stimulus for each location of photostimulation. The mean current amplitude of the intracortical responses was measured in a window of 100 ms, 7 ms after the onset of the photostimulus to avoid contamination due to evoked direct responses. Typically two to four maps were obtained per cell and averaged. These averaged single-cell maps were used to compute group-averaged maps. For display only, interpolation was performed on averaged synaptic input maps.

The input domains in thalamus were generated based on interpolated synaptic input maps of the cortical cells. For each cell, synaptic responses evoked in all trials were expressed as a ratio of the largest response. The contours are the isolines of inputs that are 50% of the largest response.

The mean distance of L4, L5A, or L2/3 synaptic inputs feeding into L2 and L3 was calculated as Σ(synaptic input × absolute lateral distance from the center of barrel or septum)/Σ(synaptic input). The mean distance from the soma of L4 input feeding into L3 cells was calculated as Σ(synaptic input × absolute lateral distance from the soma)/Σ(synaptic input).

Traces of loose-seal recordings were analyzed for APs. A spatial profile of excitability (excitation profile) was generated by color coding the number of APs elicited at each uncaging site within 100 ms after the stimulus. The mean distance from the soma at which APs were evoked was calculated as Σ(APs × absolute distance from the soma)/ΣAPs). In barrel cortex, APs were elicited 37 ± 4 μm laterally and 37 ± 5 μm vertically from the soma of L4 stellate cells (*n =* 16). APs were elicited 33 ± 8 μm laterally and 38 ± 13 μm vertically from the soma of L5A pyramidal cells (*n =* 4). Under our conditions, LSPS excites neurons in the top 100 μm of the brain slice [13].

All data are presented as an average ± standard error of the mean (s.e.m.).

## Supporting Information

Figure S1 Excitation Profiles of L4 and L5A Cells (A) Average excitation profile of L4 cells located close to the L4/L5A boundary. Photostimulation over L5A rarely elicited APs in these cells. Pixel values encode the number of APs in a 100-ms window after photostimulation.
(B) Average excitation profile of L5A cells located close to the L4/L5A boundary. These neurons were all pyramidal. Photostimulation over L4 readily excited these cells.
(687 KB EPS)

Figure S2 Input Ratios for Groups of L2/3^Barrel^ Cells in the Same Slice (A) Schematic showing the regions of interest used in (B).
(B) The ratios of input from different layers as a function of distance of L2^Barrel^ (closed circles) and L3^Barrel^ (open circles) cells from the pia. Groups of cells tested in the same slice are shown with symbols of the same colors.
(445 KB EPS)

Figure S3 Center of Mass of Synaptic Inputs (A) Schematic of the analysis shown in (B). The distance (d) between the soma (circle) and the center of mass (cross) of the L4 projections was measured for individual L3 cells. The center of mass is: Σ(synaptic input × lateral distance from soma)/Σ(synaptic input).
(B) Distance (d) between the center of mass of the L4 (green) and L5A (blue) projections, and the somata of L3 and L2 cells located above barrels and septa. Open circles are for individuals cells, closed circles are the averages ± s.e.m.
(520 KB EPS)

Table S1 Properties of Photostimulation-Evoked Synaptic Responses in Cortical Neurons and Firing in Thalamus (a) The onset for the largest response for each cortical cell. The onset is the time between UV stimulus and when the EPSC crosses a threshold, defined as 4× the standard deviation in a control baseline period.
(b) Largest response. The response is the EPSC averaged over 100 ms after the stimulus.
(c) and (d) At a particular map location, synaptic responses across trials were aligned at their onset and averaged. The 20%–80% rise time (c) was measured for isolated EPSCs and for the first EPSC in bursts (time between first and second EPSC, >7 ms). The 20%–80% decay time (d) was measured for isolated EPSCs only. EPSCs at thalamocortical synapses had significantly slower kinetics in L5A cells compared to L4/5B/6A cells. An asterisk (*) indicates *p* < 0.001 (Wilcoxon).
(e) and (f) The onset of the first AP (e) and the total number of APs (f) evoked by photostimulation in a 8 × 8 grid centered on soma was measured in VPM and POm cells.
Numbers in parenthesis indicate the number of cells.
(44 KB DOC)

## Abbreviations

AP: action potential
L: layer
EPSC: excitatory postsynaptic current
LSPS: laser scanning photostimulation
POm: posterior medial nucleus of thalamus
UV: ultraviolet
VPM: ventral posteromedial nucleus of thalamus
