# Correction: Interdigitated Paralemniscal and Lemniscal Pathways in the Mouse Barrel Cortex

**DOI:** 10.1371/journal.pbio.0050028

**Published:** 2007-01-16

**Authors:** Ingrid Bureau, Francisca von Saint Paul, Karel Svoboda

In *PLoS Biology*, volume 4, issue 12: doi:10.1371/journal.pbio.0040382

Because of an editorial error, the author contributions were printed incorrectly. The correct author contributions are printed below.

**Author contributions.** IB and KS conceived and designed the experiments. IB and FvSP performed the experiments. IB and KS analyzed the data. IB and KS wrote the paper.

