# Supplementary material for: Interdigitated Paralemniscal and Lemniscal Pathways in the Mouse Barrel Cortex
Source: PLoS Biol. 2006 Nov 21;4(12):e382. doi: 10.1371/journal.pbio.0040382 (PMC1637129; doi:10.1371/journal.pbio.0040382)
Supplement: Supplementary file 1 [file pbio.0040382.st001.doc]

**SUPPLEMENTAL TABLE 1**

| **CORTICAL LAYER** | **THALAMOCORTICAL RESPONSE PROPERTIES** | |
| --- | --- | --- |
|  | **Onset of largest response (ms) (a)** | |
|  | Mean ±  sem | Range |
| **L4**  **L5A**  **L5B**  **L6A** | 13 ± 6 (28)  25 ± 2 (42)  16 ± 3 (13)  17 ± 8 (6) | 5 - 58  4 - 49  6 - 44  7 - 55 |
|  | **Largest response (pixel value) (pA) (b)** | |
|  | Mean ± sem | Range |
| **L4**  **L5A**  **L5B**  **L6A** | 10 ± 1 (28)  8 ± 1 (42)  8 ± 1 (13)  5 ± 2 (6) | 3 - 33  3 - 25  3 - 18  3 - 13 |
|  | **20-80 % rise time single EPSC (ms) (c)** | |
|  | Mean ± sem | Range |
| **L4**  **L5A**  **L5B**  **L6A** | 1.3 ± 0.1 (13)  1.9 ± 0.2 * (18)  1.2 ± 0.1 (9)  0.9 ± 0.1 (3) | 0.5 - 1.8  1.2 - 3.8  0.8 - 1.9  0.8 - 1.0 |
|  | **20-80 % decay time single EPSC (ms) (d)** | |
|  | Mean ± sem | Range |
| **L4**  **L5A**  **L5B**  **L6A** | 10.1 ± 1.4 (4)  14.1 ± 1.3 * (10)  6.6 ± 0.3 (3)  7.5 ± 2.7 (2) | 7.3 - 13.8  9.9 - 23.5  6.0 - 6.9  4.8 - 10.1 |

| **THALAMIC NUCLEUS** | **UNCAGING-EVOKED FIRING PROPERTIES** | |
| --- | --- | --- |
|  | **Onset (ms) (e)** | |
|  | Mean ± sem | Range |
| **VPM**  **POm** | 16 ± 3 (7)  23 ± 3  (7) | 3 - 55  6 - 66 |
|  | **Total number of APs in 8×8 grid (f)** | |
|  | Mean ± sem | Range |
| **VPM**  **POm** | 6.1 ± 1.4 (7)  5.4 ± 1.6 (7) | 2 - 13  2 - 13 |
